# Ex vivo apoptotic and autophagic influence of an estradiol analogue on platelets

**DOI:** 10.1186/s40164-016-0048-z

**Published:** 2016-07-15

**Authors:** Lisa Repsold, Etheresia Pretorius, Annie Margaretha Joubert

**Affiliations:** Department of Physiology, Faculty of Health Sciences, School of Medicine, University of Pretoria, Pretoria, South Africa

**Keywords:** Platelets, Ex vivo, 2-ethyl-3-*O*-sulphamoyl-estra-1,3,5(10)16-tetraene, Apoptosis, Autophagy

## Abstract

**Background:**

Platelets are known contributors to the vascularization, metastasis and growth of tumors. Upon their interaction with cancer cells they are activated resulting in degranulation and release of constituents. Since the apoptotic- and autophagic effects of 2-ethyl-3-*O*-sulphamoyl-estra-1,3,5(10)16-tetraene (ESE-16) has been shown to occur in vitro and this compound was designed to bind to carbonic anhydrase II (CAII), the possible occurrence of these cell death mechanisms in platelets as circulatory components, is of importance.

**Methods:**

Scanning electron microscopy was used to assess morphological changes in platelets after exposure to ESE-16. The possible apoptotic- and autophagic effect of ESE-16 in platelets was also determined by means of flow cytometry through measurement of Annexin V-FITC, caspase 3 activity, autophagy related protein 5 levels and light chain 3-I to light chain 3-II conversion.

**Results:**

Scanning electron microscopy revealed no changes in ESE-16-treated platelets when compared to vehicle-treated samples. Apoptosis detection by Annexin V-FITC and measurement of caspase 3 activity indicated that there was no increase in apoptosis when platelets were exposed to ESE-16. The incidence of autophagy by measurement of autophagy related protein 5 levels and light chain 3-I to light chain 3-II conversion showed that exposure to ESE-16 did not cause the incidence of autophagy in platelets.

**Conclusion:**

This is the first ex vivo study reporting on involvement of apoptosis- and autophagy-related targets in platelets after exposure to ESE-16, warranting further investigation in platelets of cancer patients.

## Background

Apoptosis is a form of cell death, which is closely associated with occurrences within the nucleus, and is consequently questioned in platelets since they lack the nuclear machinery typically considered to result in apoptosis. Interestingly, platelets display characteristic signs of nucleated apoptosis including membrane blebbing, loss of the integrity of the platelet membrane and microparticles release [1, 2]. The ability of platelets to undergo apoptosis is a result of the presence of mitochondria which contribute mitochondrial deoxyribonucleic acid (DNA) and messenger ribonucleic acid (mRNA) which aid in the platelets’ ability to synthesise proteins contained within platelet granules [3, 4].

Thus, even though platelets do not possess a nucleus, they exhibit biological apoptotic signals during stress conditions including activation of caspase 3 and exposure/externalisation of phosphatidylserine [5]. Kile [6] showed that platelets do undergo apoptosis via the intrinsic apoptotic pathway, which also regulates the platelets’ lifespan. Platelets abundant mitochondria are known to be associated with the intrinsic apoptotic pathway or mitochondrial pathway [7].

The intrinsic apoptotic pathway is characterised by activation of Bak and Bax, triggering damage of the mitochondria and releasing cytochrome *c* and other apoptotic proteins from the mitochondrial intermembrane space. The release of cytochrome *c* allows for the formation of the apoptotic protease activating factor 1 (Apaf-1) apoptosome and subsequent recruitment of initiator procaspase 9. Binding to the apoptosome activates caspase 9 and resulting activation of effector caspase 3, resulting in the execution phase of apoptosis [6]. Upstream of caspase 3 activation and phosphatidylserine (PS) exposure, the mitochondrial inner transmembrane potential is depolarized in platelets, similar to the mechanism of nucleate cellular apoptosis [3].

The resulting externalisation of PS allows for removal of apoptotic platelets. In platelets, PS is also expressed on the cell surface, however, can only be recognized by macrophages for phagocytosis by recognition via human cluster of differentiation 36 (CD36) present on the membrane of human platelets [3, 5–7]. The externalisation of PS in platelets, however, seems to also occur independently of the intrinsic apoptotic pathway, playing an important role in formation of thrombin by assembling the pro-thrombinase complex [6, 7].

In addition to apoptosis in platelets, the role of autophagy and the biological markers thereof have not been researched extensively in platelets. Since platelets do contain small amounts of functional mitochondria, it has been proposed to share characteristics of nucleated autophagy mechanisms and markers [8]. Autophagy’s ability to maintain cellular homeostasis and adjustment to starvation is of importance in platelets since their lifespan is only about 10 days in humans [9]. However, autophagy can also be triggered continuously under certain stress conditions such as starvation, cellular injury and contact with certain chemicals, which lead the cell to progressively degrade vital cytoplasmic components, essentially digesting itself [9].

The occurrence of autophagy is not well documented in platelets; literature reveals a research gap in determining whether autophagy occurs in platelets and by which mechanisms it is regulated. One such a study was conducted by Feng et al., where researchers showed that platelets do express autophagy-related gene (Atg) proteins and the process is also activated by the inhibition of mammalian target of rapamycin (mTOR) [10]. The occurrence of autophagy in platelets is essential in maintaining homeostasis within platelets and in the number of platelet populations [10]. A defect in platelet autophagy may result in compromised platelet adhesion and aggregation [10].

Platelets also serve as way for tumors to increase growth and provide physical and mechanical support to elude the immune system and metastasize [11, 12]. Due to the fact that platelets play an important role in cancer and tumor development, the effect of potential anticancer drugs on platelets will provide research data regarding its role in cancer progression.

Cancer metastasis is directly linked to platelet activity and in particular the ability of cancer cells to elude the immune system by formation of platelet-tumor aggregates [13]. This takes place through the binding of cancer cells (from various cancer cell lines) to P-selectin and integrins expressed on the membrane of platelets thus activating them.

Testing of newly synthesized compounds on cancer cell lines allows for the opportunity to determine the mechanisms of action and the possible effects and success of these compounds as potential anticancer compounds. Our laboratory has determined the aforementioned effects of ESE-16 on cancer cell lines including tumorigenic human epithelial cervical HeLa cell line, MCF-7 breast cancer cell line, esophageal carcinoma SNO cell line and the metastatic MDA-MB-231 breast cancer cell line, whilst also being tested on the non-tumorigenic MCF-12A breast cells to assure cancer cell selectivity [14–16]. However, since ESE-16 was designed to reversibly bind to CAII in the blood stream to bypass the first pass of metabolism in the liver, thereby increasing its bioavailability, its resulting effects on blood components is of extreme importance [17]. Platelets, in particular, present with specific characteristics making them a target for cancer studies [18].

The ex vivo effect of ESE-16 on platelets and possible instigation of apoptosis and autophagy have not been reported in literature. We investigated the role platelets may play after exposure to ESE-16 and how exposure to ESE-16 will influence apoptosis and thus the incidence of externalisation of PS and caspase 3 in healthy human platelets. Results provide with substantial evidence that future in vivo studies with ESE-16 are plausible and that this compound has beneficial potential in the field of cancer research.

## Materials and methods

### Materials

Citrate tubes (with citrate as anticoagulant causing chelation of extracellular calcium [19]) and needles were acquired from transpharm (Gauteng, SA). Microplates (96 well) were obtained from Separation Scientific (Randburg, Johannesburg, SA). Phosphate-buffered saline (PBS) was purchased from Gibco-BRL (Invitrogen, Carlsbad, CA, USA) and prepared as a tenfold concentrated stock solution consisting of 80 g/l NaCl, 2 g/l KCl, 2 g/l KH_2_PO_4_ and 11.5 g/l Na_2_PO_4_. The latter was prepared in double distilled water (ddH_2_O) and the pH was adjusted to 7.4. A one times solution of PBS was made with ddH_2_O as a 1:10 dilution of the tenfold stock and subsequently autoclaved (120 °C, 15 psi, 20 min) before use. Dimethyl sulphoxide (DMSO) and propidium iodide were supplied by Sigma-Aldrich Co. (St. Louis, USA). All blood contaminated waste materials were collected and discarded into 5L Biological waste bins (Sharps bin). Bins were handed over to Oricol Environmental Services.

#### Preparation of compounds

ESE-16 is a novel analogue of 2-methoxyestradiol which has been in silico-designed at the Bioinformatics and Computational Biology Unit, Department of Biochemistry, University of Pretoria, South Africa. Synthesis of ESE-16 was conducted by Ithemba Pharmaceuticals (Pty) Ltd and ESE-16 was dissolved in dimethyl sulfoxide of which the final concentration did not exceed 0.01 % (v/v) in platelet samples [20]. Platelet samples were exposed to 0.20 μM of ESE-16 [17] for 24 h at 22 °C which was selected as it has been previously established that 0.18 μM ESE-16 inhibits cell growth by 50 % (GI_50_) after 24 h at 37 °C [17, 21, 22]. Platelet samples were exposed for 24 h at 22 °C as this temperature has been determined to be comparable to that of fridge temperatures for platelet storage in order to maintain platelet viability [1]. Controls included DMSO at a concentration of 0.01 % as vehicle control (v/v), untreated platelets as negative control, 2-methoxyestradiol-bis-sulphamate-treated (2MEBM) platelets at a concentration of 1 µM and platelets treated with 4 % DMSO as positive controls [23].

### Methods

#### Study design and sampling method

Blood was collected from 3 healthy female individuals working at the Department of Physiology (University of Pretoria) and aged between 20 and 45 who did not smoke or use any medication. Participants who met the following exclusion criteria were involved in the study: chronic or acute illnesses, autoimmune diseases, hereditary diseases, hypertension, contraceptives, or smoking. As breast cancer is the second leading cause of mortality of females globally and first leading cause in sub-Saharan Africa with poor survival rates, female participants were chosen for this study, to determine possible future clinical applications [24, 25]. Even though the compounds tested in this study are derived from 17β-estradiol, the compounds do not bind selectively to estrogen receptors, yet rather to CAII [17, 21, 22].

Blood samples were taken after an 8 h period of fasting between 08:00 and 09:00 am. Whole blood was collected in citrate tubes and platelet rich plasma (PRP) was obtained by centrifuging the blood at 1000 rpm for 2 min and collecting plasma from the separated blood [26]. The PRP was centrifuged further, supernatant was discarded and platelets were resuspended in plasma.

#### Scanning electron microscopy

The scanning electron microscope (SEM) is used to view the surface and surface molecules of samples with the use of high-energy electrons which reflected off the surface of the solid coated specimen surface to present a high-quality image [27]. Morphology of platelet samples exposed to ESE-16 at 24 h to determine effects at this time interval equivalent to exposure of cancer cell lines at 22 °C was viewed.

Ex vivo samples were prepared on the glass plates with 10 µl platelets (10^7^ platelets/ml) as a control, 10 µl platelets exposed to ESE-16, 10 µl platelets exposed to DMSO and 10 µl platelets exposed to positive control 2-methoxyestradiol-bis-sulphamate and 4 % DMSO. Glass plates with ex vivo samples were placed in 6 well plates and left to dry slightly, after which the samples were washed for 20 min in a 50 % PBS: 50 % distilled H2O solution. Samples were fixed with 2.5 % gluteraldehyde and PBS for 30 min and washed 3 times in PBS for 3 min each for subsequent secondary fixation in osmium tetraoxide for 15 min. Samples were washed 3 times each for 3 min and dehydrated for 3 min each in increasing concentration of ethanol, 30, 50, 70, 90 % and three times in 100 % ethanol [26]. Samples were critically dried, mounted and carbon coated and viewed with the Zeiss ULTRA plus FEG-SEM [Carl Zeiss (Pty) Ltd, Johannesburg, South Africa]. Qualitative SEM images were obtained from 3 independent experiments repeated for each participant. Representative images were included to represent all 3 repeats.

#### Measurement of phosphatidyl-serine (PS) flip via annexin V-fluorescein isothiocyanate

During apoptosis, activation of calcium-dependent phospholipid scramblase causes the symmetry of the phospholipid content of the cell membrane to be lost [28]. This occurs in platelets following activation and results in the externalization of the phospholipid layer which can be detected using the PS-binding protein, annexin V [29].

Platelets were obtained and exposed as previously described. Platelet samples were resuspended in 100 µl of the 1 × binding buffer. Subsequently, 10 µl of annexin V-fluorescein isothiocyanate (FITC) was added and incubated for 15 min in the dark at room temperature as described in supplier manual from MACS (Miltenyi Biotec GmbH) [29]. After incubation, samples were washed with 1 ml 1 × binding buffer and centrifuged at 300×*g* for 10 min. The supernatant was carefully pipetted off and samples were resuspended in 500 µl 1 × binding buffer solution. Annexin V fluorescence was measured with a FC500 System flow cytometer equipped with an air-cooled argon laser with an excitation wavelength of 488 nm. 10,000–30,000 events were counted for each repeat and analysis of the data was done with the use of Cyflogic version 1.2.1 software.

#### Measurement of caspase 3 activity

Caspase 3, an execution caspase in the apoptotic pathways and upon activation, cleaves various proteins which results in the characteristic qualities of apoptosis including formation of apoptotic bodies [30]. Activation of caspase 3 due to ESE-16-exposure was investigated with the use of flow cytometry [31].

Samples were washed in wash buffer and centrifuged at 250×*g* for 5 min and the pellet was fixed by the addition of fixation buffer (0.1 % formaldehyde) and incubated at room temperature for 20 min. Samples were then centrifuged at 10,000×*g* for 1 min and supernatant was discarded. Cells were washed once in assay buffer [containing 1 % bovine serum albumin (BSA)] and centrifuged. Samples were suspended in 500 µl cold Permeabilization buffer (100 % methanol) and incubated on ice for 10 min. Cells were centrifuged, supernatant discarded and washed in assay buffer. Samples were spun down, suspended in 100 µl Assay buffer with a 1:100 dilution of the primary antibody, rabbit anti-active caspase 3 [IMGENEX (San Diego, California, USA) purchased from BIOCOM Biotech (Pty) Ltd. (Pretoria, Gauteng, South Africa)] and incubated for 90 min on ice, after which 900 µl Assay buffer was added to wash the samples. Samples were subsequently washed twice with 500 µl Assay buffer, spun down, resuspended in 100 µl Assay buffer and incubated for 1 h in the dark with 0.2 µg/ml of the secondary antibody, anti-rabbit antibody conjugated to Dylight™ 488 fluorochrome [Rockland Inc. (Gilbertsville, Pennsylvania, USA) purchased from BIOCOM Biotech (Pty) Ltd. (Pretoria, Gauteng, South Africa)]. Afterwards 900 µl of the Assay buffer was added to wash the samples, centrifuged and washed twice more with 500 µl assay buffer. Cells were spun down and resuspended in 500 µl of Assay buffer. Fluorescence was measured of the FL1 channel with flow cytometry.

#### Measurement of autophagy related gene 5

The Atg5 protein is a gene product which is necessary for the formation of autophagosomes and is thus required to activate autophagy and enhances susceptibility towards apoptotic cell death [32].

Samples were washed and pelleted at 1000 × g, resuspended in 1 ml PBS containing 4 % formaldehyde and incubated at 37 °C for 10 min. Samples were left on ice for 1 min before centrifugation. Supernatant was discarded and samples were resuspended in 4 ml 100 % ice-cold methanol while slowing vortexing. Subsequently samples were incubated on ice for 30 min, after which 2 ml incubation buffer was added and samples were centrifuged and resuspended in 100 µl incubation buffer for 10 min at room temperature. Samples were stained with primary antibody cocktail (0.05 % triton X-100, 1 % BSA and 1 µg/ml anti Atg5 in PBS purchased from BIOCOM biotech Pty (Ltd) (Clubview, South Africa) and incubated at room temperature for 60 min. Samples were rinsed with incubation buffer, centrifuged at 1000×*g*, resuspended in incubation buffer and secondary antibody cocktail was added for 30 min at room temperature protected from light. Samples were washed with incubation buffer, resuspended in 0.5 ml incubation buffer and were analyzed by flow cytometry.

#### Measurement of light chain 3-II protein

The autophagy protein light chain 3 (LC3) is known to associate with the membranes of autophagomes and is essential for their formation. LC3-I is a cytosolic protein while the LC3-II protein is membrane bound, detection of the conversion of LC3-I to LC3-II is a sensitive marker for identifying autophagy in cells [9, 33].

Samples were washed with cold PBS and centrifuged at 1000×*g* to obtain a pellet. Samples were fixed with 3 ml 0.01 % formaldehyde in PBS for 10 min at 4 °C, centrifuged and was resuspended in 200 µl PBS after which the samples were incubated in 1 ml methanol (4 °C) for 15 min at 4 °C. The pellet was washed twice with cold PBS and samples were stained with 0.5 ml antibody cocktail [0.05 % triton X-100, 1 % BSA and 0.5 μg/ml conjugated rabbit polyclonal anti-LC3B antibody purchased from BIOCOM biotech Pty (Ltd) (Clubview, South Africa)] prepared in PBS for 2 h at 4 °C. Samples were washed thrice with PBS containing 0.05 % triton X-100 and 1 % BSA and were analyzed via flow cytometry.

#### Statistics

Quantitative and qualitative data were obtained. Qualitative data include SEM images and were confirmed by quantitative data. Quantitative data included flow cytometry measurement of annexin V-FITC, caspase 3, Atg5, and LC3. Samples consisted of platelets collected from 3 healthy individuals. Sample size was validated and confirmed by a biostatistician at the Research Office, Faculty of Health Sciences, University of Pretoria. Data were expressed as a ratio of the value measured for the ESE-16-treated samples compared to the vehicle-treated exposed samples defined as mean relative fluorescence. This involved flow cytometry analysis of at least 10,000–30,000 events that was repeated in triplicate (three independent experiments) whereafter a representative figure was chosen for each experiment with the use of Cyflogic version 1.2.1 software which calculates the means of X and Y co-ordinates for each treatment [17]. Statistical analysis consisted of ANOVA student’s *t* test of which a *P* value of <0.05 was considered to be statistically significant to compare the assessment results for the treatment groups and biological variations. Standard deviations were also determined and indicated in bar graphs. Statistically insignificant data obtained are of utmost importance to prove the hypothesis that ESE-16 does not cause damage to platelets per se.

## Results

### Scanning electron microscopy

**Fig. 1:**
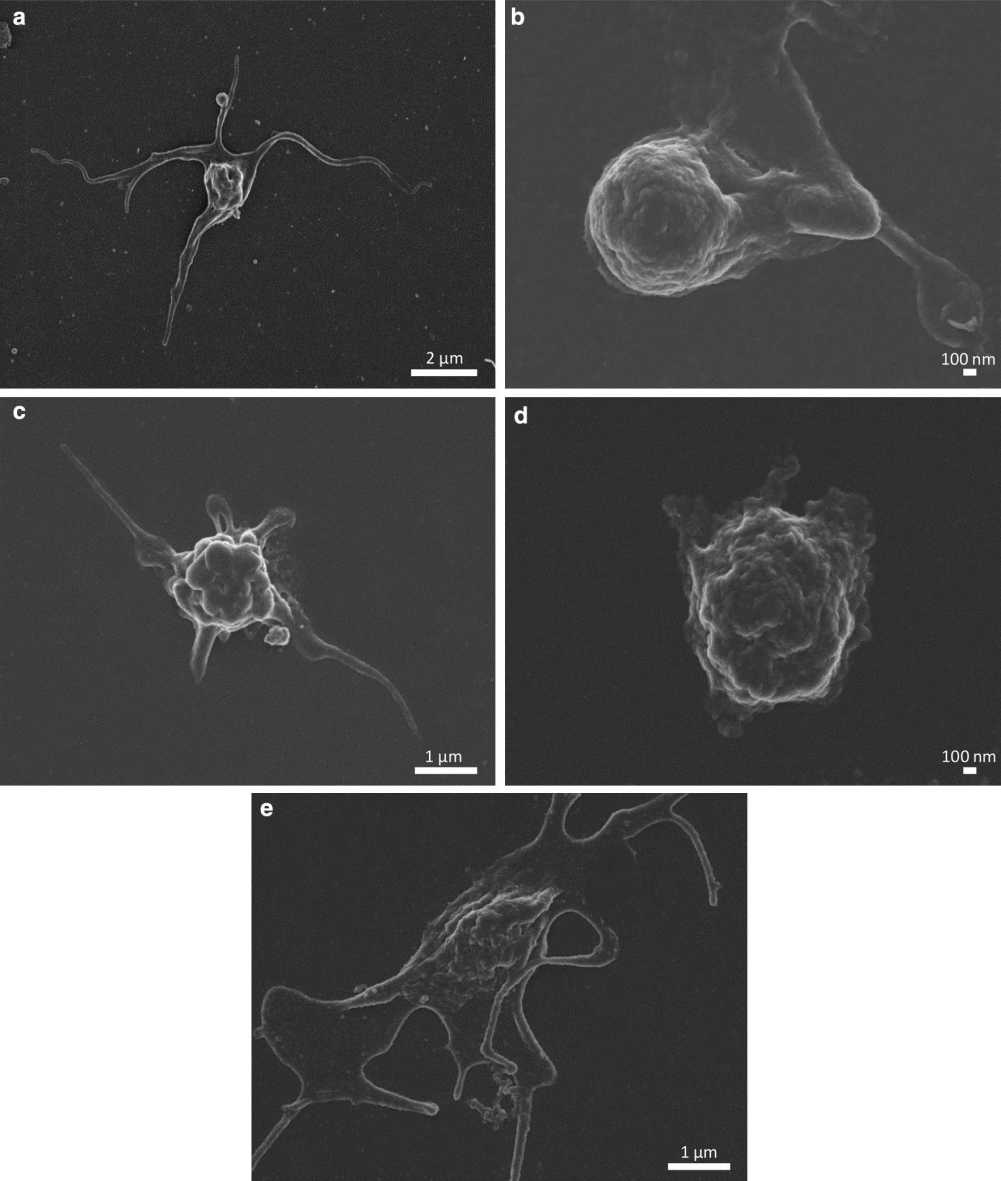
SEM images of platelets of patient 3 exposed to ESE-16 and various controls. **a** Control platelet sample indicating normal morphology of platelets. **b** DMSO-exposed samples. **c** ESE-16-treated platelets indicating that the morphology of platelets was not affected following exposure to ESE-16. **d** 2MEBM-treated platelets. **e** Platelets treated with 4 % DMSO as a positive control for platelet damage

With the use of the SEM, the surface structure and morphology of biological structures can be viewed to determine potential morphological changes [34]. SEM provides high-quality, high-resolution images of platelets exposed to ESE-16 and appropriate controls. These images are shown in Fig. 1 and indicate that platelets are not significantly activated after exposure to ESE-16. Activation of platelets is clearly shown in the positive control images (Fig. 1d, e), with severe spreading and formation of fibrin networks, indicating initiation of coagulation.

### Measurement of phosphatidyl-serine (PS) flip via annexin V-fluorescein isothiocyanate

As previously discussed, PS is externalised on platelets during platelet activation and apoptosis. Platelet apoptosis occurs through a Bak/Bax-caspase-mediated pathway independent of activation of platelets [35]. A platelet isolation and activation assay was performed (data not shown) using the human cluster of differentiation 41 (CD41) and human cluster of differentiation 62 (CD62P) markers. CD41 allows for the isolation of platelets and CD62 for further quantification of activation following exposure to ESE-16 and appropriate controls. These experiments showed that exposure of platelets to ESE-16 does not cause activation of platelets, thus any increase in PS levels is attributed to apoptosis and not activation of platelets.

**Fig. 2:**
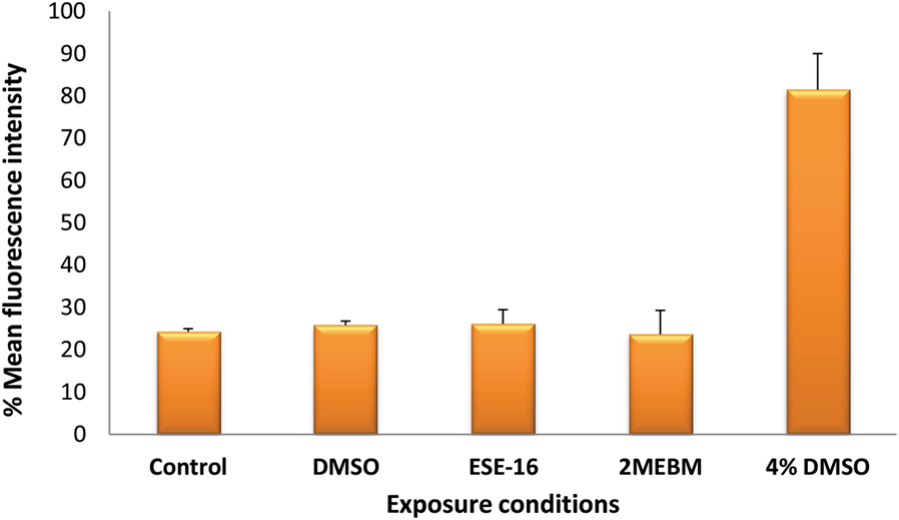
Overview bar graph of the percentage mean fluorescence intensity of apoptosis of all three patients. Exposure of platelets to ESE-16 did not cause apoptosis amongst all three patients when compared to the vehicle control- and positive control platelets (*P* value of 0.894)

The exposure of PS during platelet activation and induction of apoptosis in platelets have shown to occur in two distinctly different pathways [36]. Quantification of the extent of PS externalised in platelets is shown in Fig. 2. Mean fluorescence intensity (MFI) values indicate that the occurrence of apoptosis is not increased in a statistically significant manner in ESE-16-treated samples when compared to the vehicle control- and positive control platelets.

### Measurement of caspase 3 activity

**Fig. 3:**
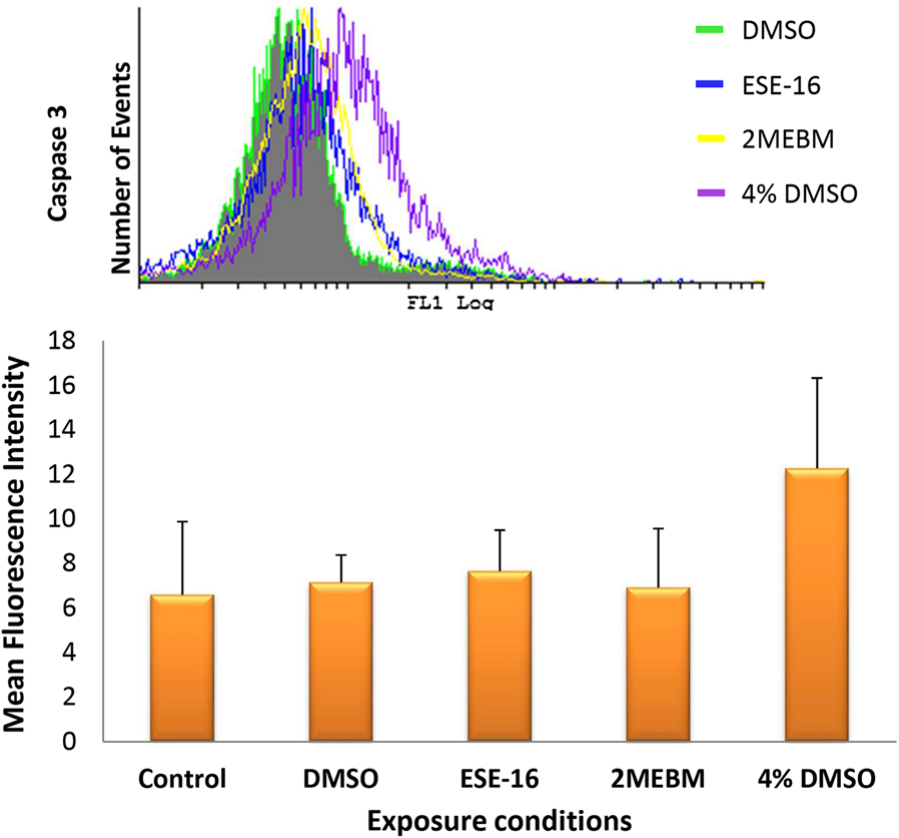
Overview histogram and bar graph of caspase 3 levels in platelets of all three patients. The vehicle control is indicated by the *green histogram*, ESE-16-treated platelets are indicated by the *blue histogram*, 2MEBM-treated samples are indicated by the *yellow histograms* and 4 % DMSO as positive control are indicated by the *purple histogram*. This overview bar graph indicates that overall the expression of caspase 3 is not increased significantly when compared to relevant controls (*P* value of 0.709)

The activation of caspase 3, an effector caspase, results in the execution phase of apoptosis [36]. The activity of caspase 3 was determined via flow cytometry after platelets were exposed to ESE-16 for 24 h. Results from three patients are indicated as an overview bar graph in Fig. 3, demonstrating that ESE-16 does not increase caspase 3 expression in platelets of patient 1–3 after exposure to ESE-16 when compared to the vehicle- and positive controls. This is shown by a *P* value of 0.539 for patient 1, 0.389 for patient 2 and 0.100 for patient 3.

### Measurement of autophagy related gene 5

**Fig. 4:**
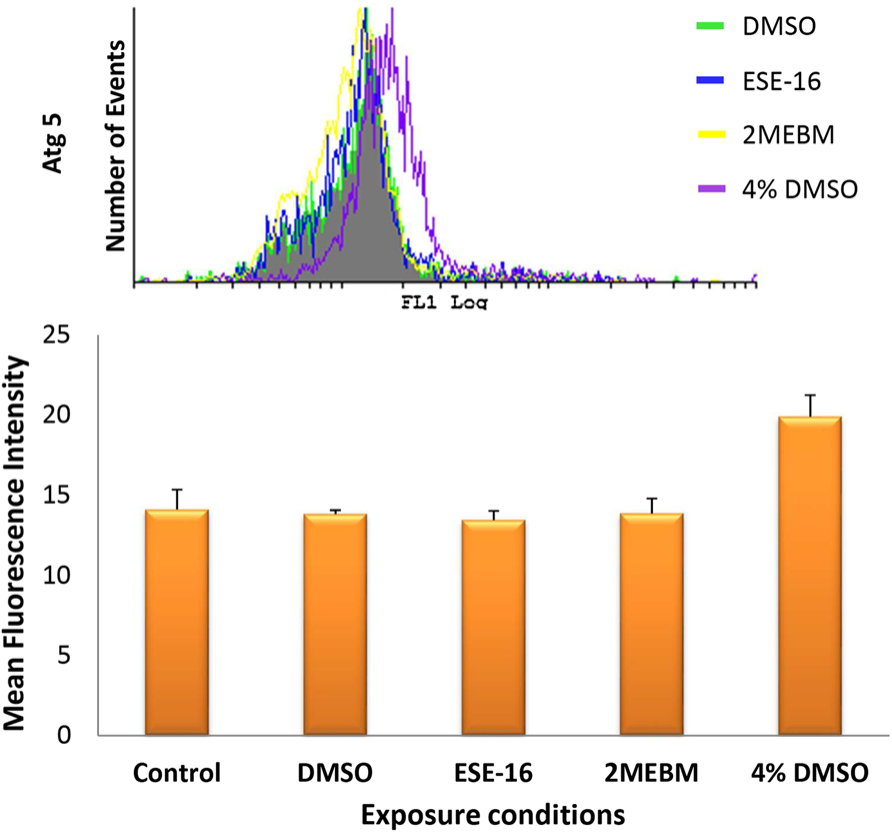
Overview histogram and bar graph of Atg 5 expression in platelets of all three patients. The vehicle control is indicated by the *green histogram*, ESE-16-treated platelets are indicated by the *blue histogram*, 2MEBM-treated samples are indicated by the *yellow histograms* and 4 % DMSO as positive control are indicated by the *purple histogram*. Atg 5 levels are not increased in ESE-16-treated platelets of all three patients (*P* value of 0.349)

The amount of Atg 5 protein expressed after exposure of platelets to ESE-16 was established via flow cytometry. This is shown in Fig. 4 for an overview bar graph of all three patients’ results. Results reveal that exposure of platelets to ESE-16 does not increase Atg 5 levels in platelets.

### Measurement of light chain 3-II protein

**Fig. 5:**
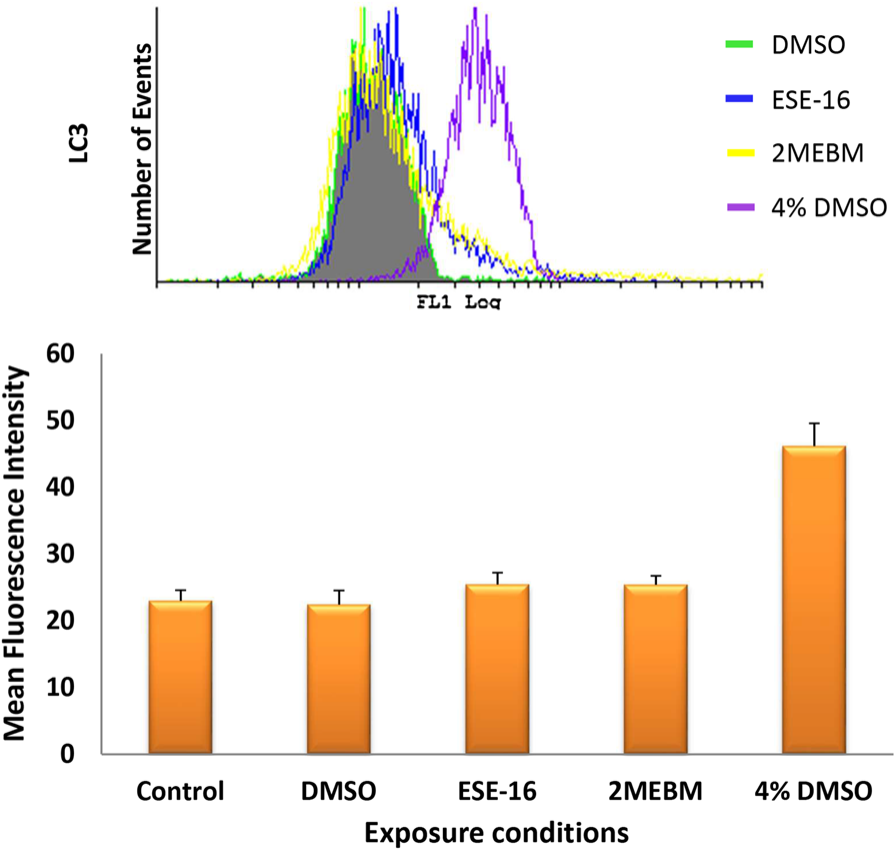
Overview histogram and bar graph indicating the average MFI of LC3 levels in platelets of all 3 patients after exposure to ESE-16 and various controls. The vehicle control is indicated by the *green histogram*, ESE-16-treated platelets are indicated by the *blue histogram*, 2MEBM-treated samples are indicated by the *yellow histograms* and 4 % DMSO as positive control are indicated by the *purple histogram*. Results show that there was a statistically insignificant difference between the vehicle control platelets and ESE-16-treated platelets between all 3 patients (*P* value of 0.134)

The conversion of LC3-I to LC3-II is an essential step in autophagy and leads to the formation of the autophagosome. LC3-II is carried on the inner- and outer membrane of the autophagosome and was quantified by means of an anti-LC3B antibody. Results from all 3 patients are illustrated in Fig. 5 as an overview bar graph showing the combined results. Results indicated that LC3 levels for all 3 patients were not increased in ESE-16-treated platelets when compared to that of the vehicle control platelets.

## Discussion

Metastatic spread is the main source of deaths related to cancer and therefore provides an important research focus in the treatment of cancer [37]. The role of platelets in cancer is crucial since they support growth and metastasis of tumors. The presence of platelet abnormalities in cancer patients has been well documented and includes the occurrence of thromboembolisms, and an increase in platelet counts, as well as platelet turnover [38].

Platelet activation by tumor cells is followed by platelet aggregation during which the invasive phenotype of tumors is enhanced by increasing the expression of matrix metallopeptidase-9 (MMP-9) and transforming growth factor β (TGFβ). Expression of MMP-9 and TGFβ in turn, activates nuclear factor-κB (NF-κB) resulting in epithelial-mesenchymal transition [39]. The effect of the novel anticancer compound, ESE-16, on platelets is thus of consequence taking into consideration its potential anticancer activity and its possible impact in cancer research.

The anticancer ability of this unique in silico-designed compound has been studied in vitro on various cancer cell lines in our laboratory [17, 21, 22]. These cell lines include the tumorigenic human epithelial cervical HeLa cell line, MCF-7 breast cancer cell line, esophageal carcinoma SNO cell line and the metastatic MDA-MB-231 breast cancer cell line [14–17, 21, 22]. Our studies confirmed that ESE-16 has similar mechanisms to that of its source compound, 2-methoxyestradiol (2ME) including tubulin disruption, inhibition of carbonic anhydrase IX (CAIX) and induction of apoptosis and autophagy [14–17, 21, 22]. In addition, it was found that the compound was more potent than 2ME in terms of antiproliferative activity at nanomolar values, while 2ME required a concentration between 1 and 2 μM to have any antiproliferative effects [17].

Promising results obtained from the in vitro studies in our laboratory subsequently advanced to ex vivo studies where the possible anti-angiogenic properties of these compounds were determined. Apoptotic- and autophagic effects have been shown to occur in vitro after exposure to ESE-16. This along with the fact that ESE-16 was designed to bind to CAII, delaying early metabolism, and is therefore carried in the circulation, signifies the importance of studying the possible occurrence of these cell death mechanisms in platelets. The effect it potentiates on blood components and especially on platelets is of significance in the study of cancer progression.

Statistically insignificant data obtained are of utmost importance in this project to prove the hypothesis that ESE-16 does not cause damage to healthy platelets *per se*. Results provide a point of reference for future investigations and since the compound does not cause morphological damage ex vivo and it does not induce apoptosis or autophagy in healthy platelets.

This research acquired substantial qualitative and quantitative data in determining the aforementioned effects. Qualitative data consisted of morphological studies pertaining to the influence of ESE-16 on the possible activation of platelets by SEM of 3 healthy females after exposure to ESE-16. Results showed no morphological changes in the platelets after exposure when compared to the vehicle control and positive control platelets. The latter control indicated severe activation with spreading of fibrin strands.

Ex vivo results are in accordance with findings by Stander et al. who suggested that ESE-16 has potential anti-CAIX activity [22]. Carbonic anhydrase (CA) is a zinc (II)-dependent enzyme which catalyses the hydration of carbon dioxide into hydrogen carbonate and a proton; thus playing an integral role in cellular fluid balance and anion-exchange systems responsible for pH and CO_2_ homeostasis [40]. CAIX contributes to the acidic extracellular environment associated with tumors which, in turn, promotes the expression of proteinases that contribute to invasion and metastasis as its expression is up-regulated under hypoxic conditions commonly found in tumors [40, 41].

ESE-16 also reversibly binds to CAII in red blood cells as previously mentioned in order to bypass the first pass metabolism in the liver which is then released into the bloodstream from CAII resulting in increased bioavailability [17]. CAII is characterized by a high affinity for sulphonamides, which could elucidate why ESE-16, a sulphamoylated analogue, does not affect platelets significantly [41]. The effect of ESE-16 on erythrocytes along with its haemolytic activity after exposure to ESE-16 has been determined and showed no significant influence, even though the compound specifically binds within erythrocytes to CAII [42, 43].

The possible occurrence of apoptosis in ESE-16-treated platelets was investigated via the annexin V-FITC apoptosis assay which quantifies the amount of externalised PS. The latter characteristic is known to occur in platelets following activation and apoptosis. Therefore, activation was first determined to ensure platelets were not activated following exposure to ESE-16 and thus not by externalised PS. Subsequently apoptosis was quantified and since activation did not occur, the extent of PS externalised was attributed to apoptosis of platelets and not activation. The MFI showed no statistically significant increase in ESE-16-exposed platelets in all 3 patients when compared to the applicable controls.

Data were corroborated with the quantification of caspase 3 levels in treated platelets. Caspase 3 is expressed in the executioner phase of apoptosis after formation of the apoptosome and activation of the initiator caspases. Levels of caspase 3 expression were not statistically significantly increased in any one of the patients when compared to the vehicle control.

Autophagy is not well documented in platelets and the presence of Atg proteins is not clearly defined in the process of autophagy in platelets. This is the first study to reveal that Atg 5 is expressed in platelets and that autophagy may occur in platelets. It is hypothesized that autophagy in platelets is of great significance as it maintains homeostasis within platelets and a fault herewith may result in deficient adhesion and aggregation of platelets [10]. Results indicate that Atg 5 is not increased in platelets in all three patients after treatment with ESE-16 when compared to the relevant controls.

The conversion of LC3-I to LC3-II takes place by means of Atg16L by conjugation to phosphatidylethanolamine that associates with the autophagosome membrane aiding in its formation [44]. The presence of this conversion to LC3-II has not been documented in platelets and with this study we demonstrated that LC3-II is present in platelets. However, LC3-II levels were not statistically significantly increased in platelets after exposure to ESE-16.

This ex vivo study is the first to investigate the unique in silico-designed compound, ESE-16’s apoptotic- and autophagic effects on platelets of healthy individuals. The compound does not cause morphological damage ex vivo and it does not induce apoptosis or autophagy in platelets of healthy individuals. In addition, ESE-16 decreased the expression of angiogenic markers including vascular endothelial growth factor warranting further ex vivo studies on angiogenic-, apoptotic- and autophagic- targets of cancer patients’ platelets.

## Conclusion

Platelets play a crucial role in tumour development. In this study the ex vivo influence of a newly, in silico-designed potential anti-cancer compound namely 2-ethyl-3-*O*-sulphamoyl-estra-1,3,5(10)16-tetraene (ESE-16) was assessed on morphology of participants’ platelets and ESE-16’s possible apoptotic- and autophagic effect by means of flow cytometry through measurement of annexin V-FITC, caspase 3 activity, autophagy-related protein 5 levels and light chain 3-I to light chain 3-II conversion.

This is the first ex vivo study to highlight possible involvement of apoptosis and autophagy in platelets after exposure to this potential anti-cancer compound warranting further investigation concerning these cell death signaling pathway targets on platelets of cancer patients.

## Abbreviations

2ME: 2-Methoxyestradiol
2MEBM: 2-Methoxyestradiol-bis-sulphamate
Apaf-1: apoptotic protease activating factor 1
Atg: autophagy-related genes
BSA: bovine serum albumin
CA: carbonic anhydrase
CAII: carbonic anhydrase II
CAIX: carbonic anhydrase IX
CD: cluster of differentiation
CO_2_: carbon dioxide
ddH_2_O: double distilled water
DMSO: dimethyl sulphoxide
DNA: deoxyribonucleic acid
ESE-16: 2-Ethyl-3-*O*-sulphamoyl-estra-1,3,5(10)16-tetraene
FITC: fluorescein isothiocyanate
HeLa: Henrietta Lacks
LC3: light chain 3
MCF-7: Michigan Cancer Foundation-7
MFI: mean fluorescence intensity
MMP-9: matrix metallopeptidase-9
mRNA: messenger ribonucleic acid
mTOR: mammalian target of rapamycin
NF-κB: nuclear factor-κB
PBS: phosphate buffered saline
PRP: platelet rich plasma
PS: phosphatidylserine
SEM: scanning electron microscopy
TGFβ: transforming growth factor β

## References

[1] Li J, Xia Y, Bertino AM, Coburn JP, Kuter DJ (2000). The mechanism of apoptosis in human platelets during storage. Transfusion.

[2] Rendu F, Brohard-Bohn B (2001). The platelet release reaction: granules’ constituents, secretion and functions. Platelets.

[3] Leytin V, Freedman J (2003). Platelet apoptosis in stored platelet concentrates and other models. Transfus Sci.

[4] Reed GL. Platelet secretory mechanisms. In: Seminars in thrombosis and hemostasis. New York City: Thieme Medical Publishers; 2004. p. 441–450. 10.1055/s-2004-833479 15354265

[5] Bertino AM, Qi XQ, Li J, Xia Y, Kuter DJ (2003). Apoptotic markers are increased in platelets stored at 37 C. Transfusion.

[6] Kile BT (2009). The role of the intrinsic apoptosis pathway in platelet life and death. J Thromb Haemost.

[7] Zhao L, Zhang W, Chen M, Zhang J, Zhang M, Dai K (2013). Aspirin Induces platelet apoptosis. Platelets.

[8] Zharikov S, Shiva S (2013). Platelet mitochondrial function: from regulation of thrombosis to biomarker of disease. Biochem Soc Trans.

[9] Hait WN, Jin S, Yang JM (2006). A matter of life or death (or both): understanding autophagy in cancer. Clin Cancer Res.

[10] Feng W, Chang C, Luo D, Su H, Yu S, Hua W, Chen Z, Hu H, Liu W (2014). Dissection of autophagy in human platelets. Autophagy.

[11] Jain S, Harris J, Ware J (2010). Platelets: linking hemostasis and cancer. Arterioscler Thromb Vasc Biol.

[12] Trikha M, Zhou Z, Timar J, Raso E, Kennel M, Emmell E, Nakada MT (2002). Multiple roles for platelet GPIIb/IIIa and alphavbeta3 integrins in tumor growth, angiogenesis, and metastasis. Cancer Res.

[13] Sharma D, Brummel-Ziedins KE, Bouchard BA, Holmes CE (2014). Platelets in tumor progression: a host factor that offers multiple potential targets in the treatment of cancer. J Cell Physiol.

[14] Nkandeu DS, Mqoco TV, Visagie MH, Stander BA, Wolmarans E, Cronje MJ, Joubert AM (2013). In vitro changes in mitochondrial potential, aggresome formation and caspase activity by a novel 17-beta-estradiol analogue in breast adenocarcinoma cells. Cell Biochem Funct.

[15] Theron AE, Nolte EM, Lafanechere L, Joubert AM (2013). Molecular crosstalk between apoptosis and autophagy indiced by a novel 2-methoxyestradiol analogue in cervical adenocarcinoma cells. Cancer Cell Int.

[16] Wolmarans E, Mqoco TV, Stander A, Nkandeu SD, Sippel K, McKenna R, Joubert A (2014). Novel estradiol analogue induces apoptosis and autophagy in esophageal carcinoma cells. Cell Mol Biol Lett.

[17] Stander A, Joubert F, Joubert A (2011). Docking, synthesis, and in vitro evaluation of antimitotic estrone analogs. Chem Biol Drug Des.

[18] Goubran HA, Burnouf T, Radosevic M, El-Ekiaby M (2013). The platelet–cancer loop. Eur J Intern Med.

[19] Wallén NH, Ladjevardi M, Albert J, Bröijersén A (1997). Influence of different anticoagulants on platelet aggregation in whole blood; a comparison between citrate, low molecular mass heparin and hirudin. Thromb Res.

[20] Joubert A, Marais S (2007). Influence of 2-methoxyestradiol on cell morphology and Cdc2 kinase activity in WHCO3 esophageal carcinoma cells. Biomed Res.

[21] Stander BA, Joubert F, Tu C, Sippel KH, McKenna R, Joubert AM (2012). In vitro evaluation of ESE-15-ol, an estradiol analogue with nanomolar antimitotic and carbonic anhydrase inhibitory activity. PLoS ONE.

[22] Stander BA, Joubert F, Tu C, Sippel KH, McKenna R, Joubert AM (2013). Signaling pathways of ESE-16, an antimitotic and anticarbonic anhydrase estradiol analog, in breast cancer cells. PLoS One.

[23] Seegers JC, de Kock M, Lottering ML, Grobler CJ, van Papendorp DH, Shou Y, Habbersett R, Lehnert BE (1997). Effects of gamma-linolenic acid and arachidonic acid on cell cycle progression and apoptosis induction in normal and transformed cells. Prostaglandins Leukot Essent Fatty Acids.

[24] Dickens C, Joffe M, Jacobson J, Venter F, Schüz J, Cubasch H, McCormack V (2014). Stage at breast cancer diagnosis and distance from diagnostic hospital in a periurban setting: a South African public hospital case series of over 1,000 women. Int J Cancer.

[25] Kamangar F, Dores GM, Anderson WF (2006). Patterns of cancer incidence, mortality, and prevalence across five continents: defining priorities to reduce cancer disparities in different geographic regions of the world. J Clin Oncol.

[26] Pretorius E (2007). The role of platelet and fibrin ultrastructure in identifying disease patterns. Pathophysiol Haemost Thromb.

[27] Goldstein J, Newbury DE, Joy DC, Lyman CE, Echlin P, Lifshin E, Sawyer L, Micheal JR (2002). Scanning electron microscopy and X-ray microanalysis.

[28] Blatt NB, Glick GD (2001). Signaling pathways and effector mechanisms pre-programmed cell death. Bioorg Med Chem.

[29] Koopman G, Reutelingsperger CP, Kuijten GA, Keehnen RM, Pals ST, Van Oers MH (1994). Annexin V for flow cytometric detection of phosphatidylserine expression on B cells undergoing apoptosis. Blood.

[30] Bao Q, Shi Y (2007). Apoptosome: a platform for the activation of initiator caspases. Cell Death Differ.

[31] Funayama E, Chodon T, Oyama A, Sugihara T (2003). Keratinocytes promote proliferation and inhibit apoptosis of the underlying fibroblasts: an important role in the pathogenesis of keloid. J Invest Dermatol.

[32] Periyasamy-Thandavan S, Jiang M, Schoenlein P, Dong Z (2009). Autophagy: molecular machinery, regulation, and implications for renal pathophysiology. Am J Physiol Renal Physiol.

[33] Gottlieb RA (2012). Autophagy in health and disease.

[34] Pretorius E (2011). Traditional coating techniques in scanning electron microscopy compared to uncoated charge compensator technology: looking at human blood fibrin networks with the ZEISS ULTRA Plus FEG-SEM. Microsc Res Tech.

[35] Schoenwaelder SM, Yuan Y, Josefsson EC, White MJ, Yao Y, Mason KD, O’Reilly LA, Henley KJ, Ono A, Hsiao S, Wilcox A, Roberts AW, Huang DC, Salem HH, Kile BT, Jackson SP (2009). Two distinct pathways regulate platelet phosphatidylserine exposure and procoagulant function. Blood.

[36] Gyulkhandanyan AV, Mutlu A, Freedman J, Leytin V (2012). Markers of platelet apoptosis: methodology and applications. J Thromb Thrombolysis.

[37] Carmeliet P, Jain RK (2000). Angiogenesis in cancer and other diseases. Nature.

[38] Sabrkhany S, Griffioen AW, Oude Egbrink MGA (2011). The role of blood platelets in tumor angiogenesis. Biochim Biophys Acta.

[39] Mannello F, Medda V (2011). Differential expression of MMP-2 and MMP-9 activity in megakaryocytes and platelets. Blood.

[40] Lloyd Matthew D, Pederick Richard L, Natesh R, Woo LWL, Purohit A, Reed MJ, Acharya KR, Potter BV (2005). Crystal structure of human carbonic anhydrase II at 1.95 A resolution in complex with 667-coumate, a novel anti-cancer agent. Biochem J.

[41] Supuran CT, Scozzafava A (2007). Carbonic anhydrases as targets for medicinal chemistry. Bioorg Med Chem.

[42] Repsold L, Mqoco T, Wolmarans E, Nkandeu S, Theron J, Piorkowski T, Du Toit P, Van Papendorp D, Joubert AM (2014). Ultrastructural changes of erythrocytes in whole blood after exposure to prospective in silico-designed anticancer agents: a qualitative case study. Biol Res.

[43] Repsold L, Pretorius E, Joubert AM (2014). An estrogen analogue and promising anticancer agent refrains from inducing morphological damage and reactive oxygen species generation in erythrocytes, fibrin and platelets: a pilot study. Cancer Cell Int.

[44] Yang ZJ, Chee CE, Huang S, Sinicrope FA (2011). The role of autophagy in cancer: therapeutic implications. Mol Cancer Ther.

